# Variations in the origin, course and branching pattern of the radial artery along with clinical correlations: narrative review

**DOI:** 10.1590/1677-5449.202400152

**Published:** 2025-03-14

**Authors:** Rajani Singh

**Affiliations:** 1 Uttar Pradesh University of Medical Sciences, Department of Anatomy, Etawah, Uttar Pradesh, India.

**Keywords:** radial artery, angiography, cannulation, artéria radial, angiografia, canulação

## Abstract

The radial artery most often arises from the brachial artery in the cubital fossa, lying deep to the brachioradialis in the upper part and superficial in the lower part. Recently, the radial artery has increasingly been used for coronary procedures. However, the radial artery is observed to vary in origin, course, and branching pattern. Precise knowledge of these parameters is essential for successful outcomes. Considering the immense clinical significance associated with this artery, this study was conducted. The aim is to expound the details of the radial artery and to highlight the associated implications, serving as a ready reference for vascular surgeons. The study was conducted using various databases and various terms related to the radial artery were used to search the literature. The literature reveals that the radial artery varies in origin, course, and branching pattern and information on these parameters will help vascular surgeons to carry out various procedures with minimum complications.

## Resumo

A artéria radial origina-se principalmente da artéria braquial na fossa cubital, situando-se profundamente ao músculo braquiorradial na parte superior e superficialmente na parte inferior. Recentemente, a artéria radial tem sido cada vez mais usada em procedimentos coronarianos. No entanto, observa-se que a artéria radial apresenta variações de origem, curso e padrão de ramificação. O conhecimento preciso dessas variações é essencial para um resultado bem-sucedido. Considerando a imensa importância clínica associada a essa artéria, o objetivo deste estudo foi descrever os detalhes da artéria radial e destacar as implicações associadas, que servirão como referência imediata para os cirurgiões vasculares. Foi realizada uma revisão da literatura em várias bases de dados, utilizando termos relacionados à artéria radial. A análise revelou que a artéria radial varia em sua origem, curso e padrão de ramificação. As informações sobre essas variações podem ajudar os cirurgiões vasculares a realizarem vários procedimentos com o mínimo de complicações.

## INTRODUCTION

**Figure 1:**
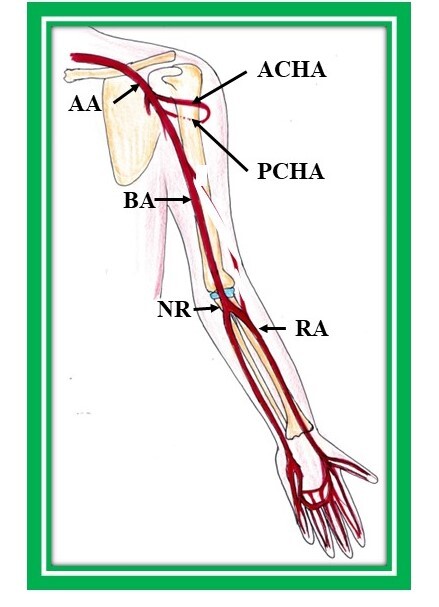
Normal origin in cubital fossa and normal course of radial artery. BA: brachial artery, NR: neck of radius, RA: radial artery, AA: axillary artery, ACHA: anterior circumflex humeral artery, PCHA: posterior circumflex humeral artery.

The brachial artery terminates into the radial and ulnar arteries at the level of the neck of the radius in the cubital fossa (Figure 1). Thus, the radial artery (RA) arises from the brachial artery as one of its terminal branches. This is the usual configuration of the RA, as described in standard anatomy textbooks. However, frequently it may originate from the axillary artery or from the brachial artery considerably above the neck of the radius bone in the arm. When the RA takes origin from the brachial artery in the arm, it is named the brachioradial artery.^1^ It descends down with lateral convexity and exits the forearm, passing posteriorly and entering the anatomical snuff box. Cranially, it is located deep to the brachioradialis muscle, but caudally it is superficial and covered only by skin and the superficial and deep fascia.^1^ The RA is the primary artery chosen for various clinical procedures such as coronary artery angiography, percutaneous coronary artery intervention, coronary artery bypass graft surgery, and cannulation, due to its easy accessibility.^2^

The RA exhibits variations in origin, course, and branching pattern. Transradial coronary artery procedures can fail in cases of abnormalities of RA in terms of origin, tortuosity, and accessory branches.^3^ In addition to this, an RA with superficial location may be mistaken for a vein and may be pierced considering it to be a vein, causing intraarterial injections and leading to clinical complications.^4^ Presently, the RA is increasingly used for coronary interventions as a substitute for the femoral artery being superficial in its course.^5^ The superficial course of the RA makes it easy to access and efficaciously squeeze for hemostasis, leading to early patient ambulation and decreased post-operative discomfort.^5^ Furthermore, it is associated with less local vascular complications in comparison to the femoral approach.^5^ In addition to this, the RA is utilized for coronary artery bypass grafting, for forearm flaps by cosmetic surgeons, and for constructing autogenous arteriovenous fistulae during renal dialysis and for vascular access for hemodialysis.^6^ Currently, increased interest in RA variations has been generated among clinicians due to its usefulness in various coronary procedures.^7^ Easy to access, increased success rate, caring for patients by nursing staff with least discomfort, and less danger of thrombosis are the chief bases for use of RA in coronary surgical interventions.^8^ The RA is intimately related to the cephalic vein, especially when it originates from the axillary artery. In this location, the RA is at risk of being damaged during intravenous injection of medications into the cephalic vein culminating in complications.^9^ Thus, abnormalities involving the RA may create complications during diagnostic, therapeutic, and surgical interventions.^10^

As is evident from the above description, comprehension of the origin, course, and branching pattern of the RA is essential for safe and successful execution of various clinical procedures. Hence this study was carried out. The aim of this study is to consolidate information and highlight implications related to the RA, serving as a ready reference for vascular surgeons to aid them in carrying out surgical procedures involving the RA uneventfully.

## MATERIAL AND METHODS

The study was conducted at the department of Anatomy, UP University of Medical Sciences, Saifai, Etawah India. Literature was explored using databases; Google scholar, Medline, Scielo, Pubmed, Scopus, Researchgate, and the Wiley online library. Standard anatomy textbooks like Gray’s Anatomy and Cunningham’s Manual of Practical Anatomy were also consulted. Only articles in English were sought. Articles containing original data were taken into account and secondary references were retrieved from bibliographies.

**Figure 2:**
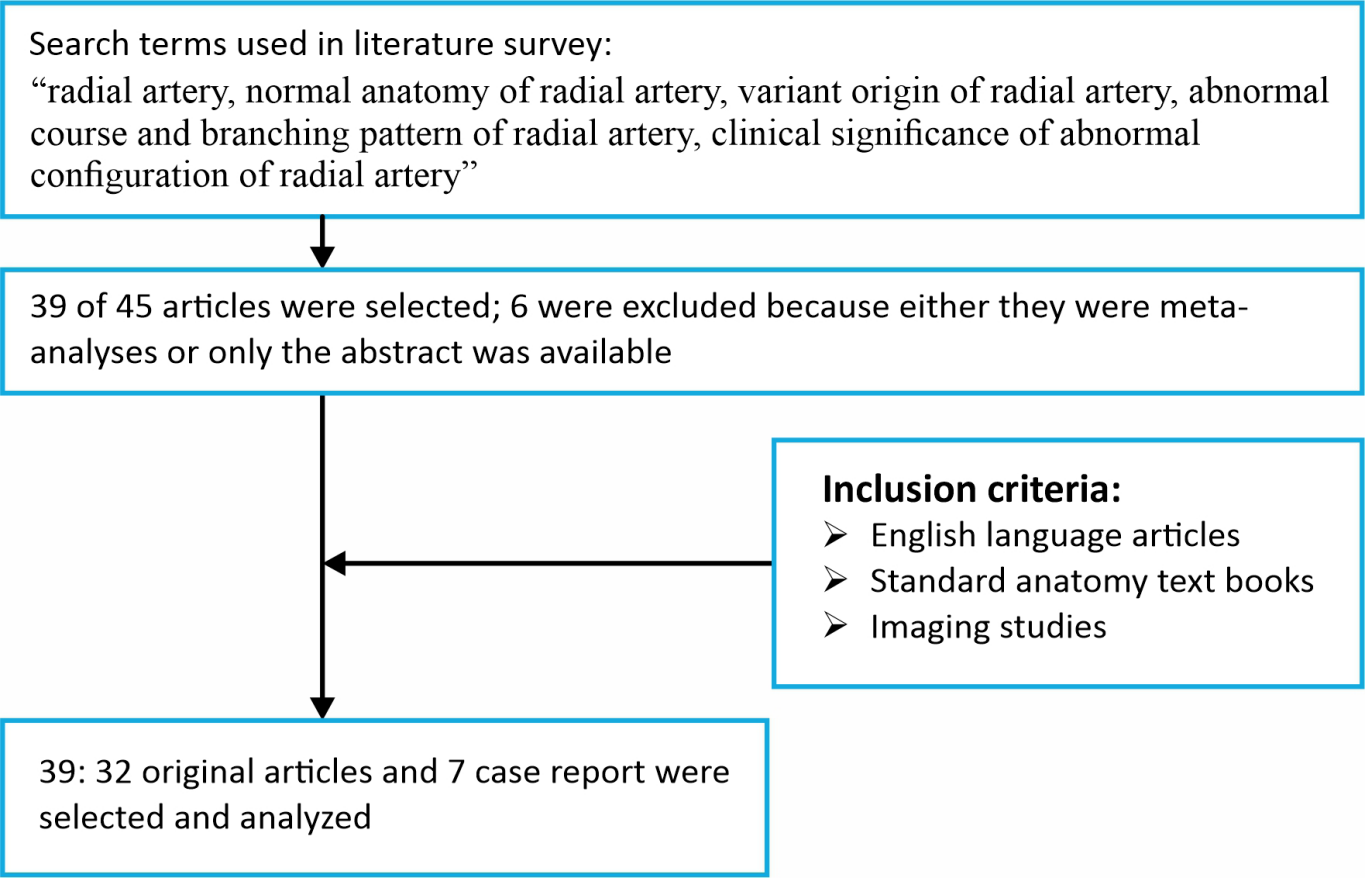
Flowchart giving brief idea of the search strategy.

Forty-five articles were taken into account. Of these, 32 full articles and 7 case report were selected for the study. Six articles were not selected because either they were meta-analyses or only the abstracts were available. A flowchart showing a summary of the search strategy is appended below (Figure 2).

Data were collected and interpreted, pitfalls during surgery related to variant configurations of the radial artery were identified, and ways to correct these pitfalls during surgical procedures were highlighted.

## RESULTS AND DISCUSSION

The RA varies in origin, course, and branching pattern. These parameters of the RA are expounded in the following paragraphs.

Deviations of configuration of the RA from standard configuration constitute the major vascular variations found in the upper limbs.^11^

The incidence of aberrant origin of the RA ranges from 4.17% to 15.6%, as described in literature.^7^ However, according to the same author,^7^ incidence is observed to be higher in angiographic studies, ranging from 8% to 24.4%. Variant and normal origins of the RA in the cubital fossa below the intercondylar line of the humerus were detected in 13.21% and 86.79% of specimens respectively.^12^

### Variant origin of the RA

**Figure 3:**
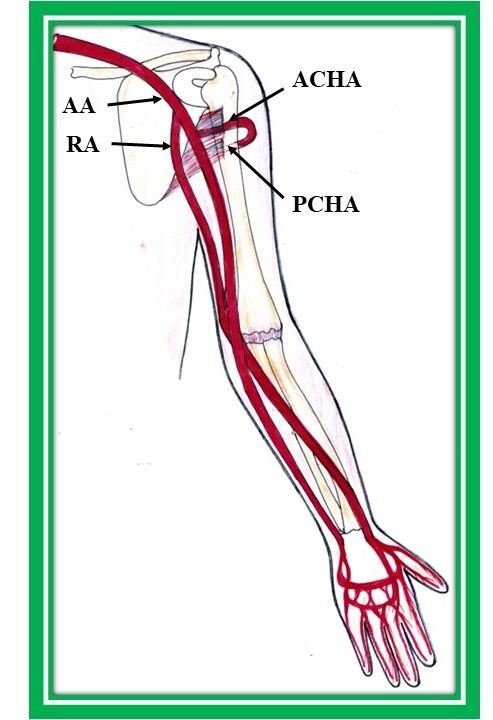
High origin of radial artery from the third part of the axillary artery. AA: axillary artery, RA: radial artery, ACHA: anterior circumflex humeral artery, PCHA: posterior circumflex humeral artery.

**Figure 4:**
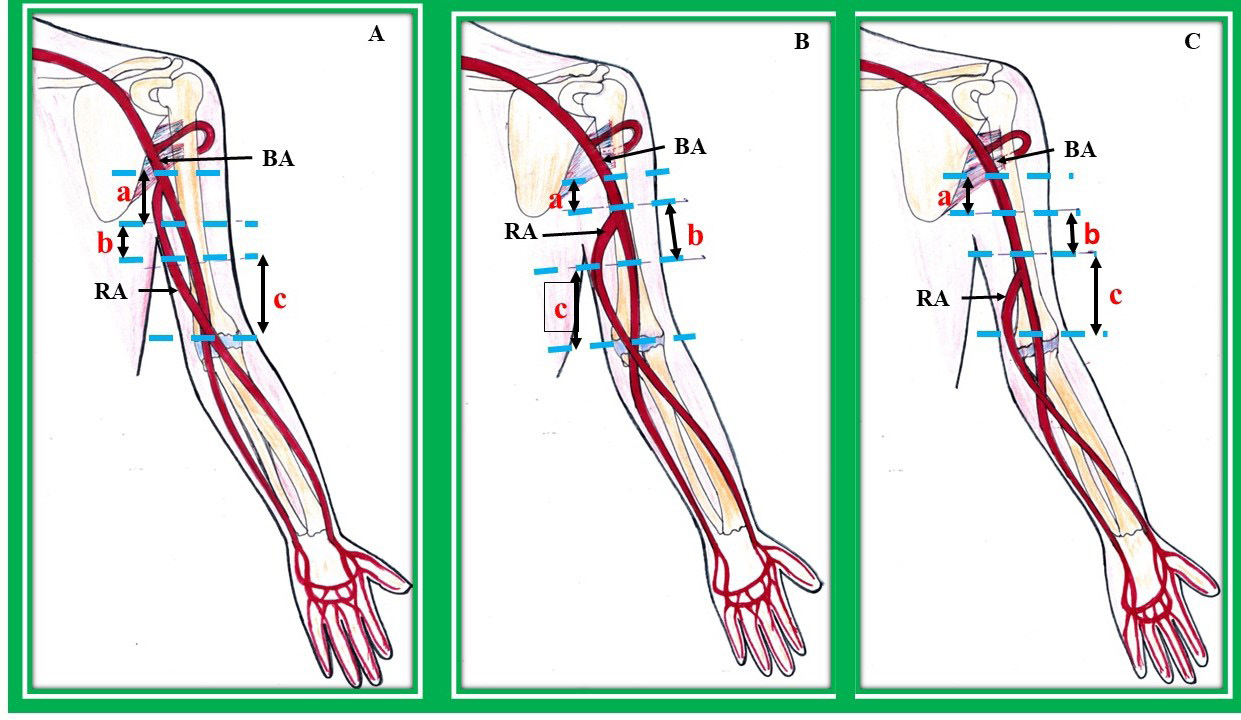
High origin of radial artery from the brachial artery in the arm. (A) Origin of radial artery from the brachial artery in the upper third or proximal arm. a- proximal arm, b- midarm, c- distal arm, BA: brachial artery, RA: radial artery; (B) origin of radial artery from the brachial artery in (b) middle third or mid arm. a-proximal arm, b-midarm, c-distal arm, BA: brachial artery, RA: radial artery; (C) origin of radial artery from brachial artery in (c) lower third or distal arm. a-proximal arm, b-midarm, c-distal arm, BA: brachial artery, RA: radial.

Origin of the RA at a higher level, from the third part of the axillary (Figure 3) or brachial artery (Figure 4A, B, and C) was the most frequent abnormality observed in the literature.^7^

The incidence rates of origin of the RA from the axillary and brachial arteries reported by various authors^1,4,6-7,12-15^ are documented in Table 1. In 1.88% of upper limbs, the RA was observed to originate from the third part of the axillary artery.^12^ Besides full text articles (Table 1),^1,4,6,7,12-15^ there are also some case reports describing the RA originating from the axillary artery, as described in the following paragraph.

**Table 1:** Displaying overall incidence of variation along with incidence rates of origin of radial artery from axillary and brachial arteries.

Investigator	No. of upper limbs	Percentage of variation	Incidence of origin of radial artery from axillary artery	Incidence of origin of radial artery from brachial artery
Haladaj et al.^1^	120	9.2	1.67	7.5
Nasr^7^	100	8	1	7
Yang et al.^4^	304	12.17	12.17	-
Kadel et al.^12^	53	13.2	1.8	11.3
Agarwal et al.^13^	32	3.12	3.12	-
Zhan et al.^14^	1200	0.25	-	0.25
Elizabeth and Vishwakarma^15^	51	7.84	1.96	5.9
Rodriguez-Niedenfuhr et al.^6^	384	36.8	3.1	13.8

There is one case report elucidating the origin of the RA from the axillary artery bilaterally.^16^ There is another case report documenting the origin of the RA from the third part of the axillary artery.^17^

The rates of variant origin of the RA are found to differ in various population.^7^ The incidence of variant origin of the RA was observed to be 5% in Africans and 2.7% in the Caucasian population,^5^ while the incidence of variant origin of the RA from the BA varied from 5.9% to 12.1% among Caucasians and was found to be 2.3% in Korean cadavers.^4^ In addition, the incidence of high origin of the RA from the BA was 0.33% among Singaporean Chinese cadavers.^18^ The cause of the existence of abnormal origin of the radial artery in various racial groups is unknown.^7^

The most common site of origin of the RA from the axillary artery is its third part. Origin of the RA from the brachial artery can be classified into three groups, as observed by various investigators.

Group I: those arising from the upper third of the arm or those arising from the proximal arm (Figure 4A);
Group II: those arising from the middle third of the arm or those arising from the midarm (Figure 4B);
Group III: those arising from the lower third of the arm or those arising from the distal arm (Figure 4C).

Rodriguez-Niedenfuhr et al.^6^ observed 384 upper limbs and designated the RA originating from the brachial artery as the brachioradial artery. The brachioradial artery was called the superficial brachioradial artery if it followed a superficial course in the forearm. As per these investigators, in 65.4% of upper limbs, the RA arose from the upper third of the brachial artery, from the middle third in 7.7%, and from the lower third in 3.9%.^6^ In another study, the incidence of origin of the RA from the upper third, middle third, and lower third of the brachial artery is reported to be 5.66%, 3.77% and 1.88%, respectively.^12^ The RA was observed to originate from the upper part of the brachial artery in 11.32%, as reported by Kadel et al.,^12^ in 8.54% by Karlsson et al.,^19^ and in 6.2% by Hassan et al.^20^

Variant origin of the RA from the axillary and brachial arteries affects the success rate of therapeutic, diagnostic, and surgical procedures and may lead to decreased success rate of the transradial approach related to coronary interventions during surgical or radiological interventions and can impair radiological interpretation.^7^ In addition to this, abnormal origins of the RA in various races may be responsible for failure of transradial interventions. The RA arising from the axillary artery or the brachial artery with a superficial course may be confused with a vein during venipuncture, leading to intraarterial injection and culminating in catastrophic hemorrhage and drug poisoning or failure of orthopedic procedures causing clinical complications.^21,22^

Therefore, information regarding the anomalous origin of the RA is essential for vascular, reconstructive, cardiac, orthopedic, or radiological manipulation.^7^

### Normal and anomalous course of the radial artery

Normally, the RA is located deep to the brachioradialis muscle in the upper forearm and becomes superficial in the lower part, covered only by skin, superficial fascia, and deep fascia, as described in standard anatomy textbooks. Deviation from this normal course of the RA in the forearm with the artery having high origin and superficial course throughout the forearm has been observed by various authors in the literature.^15,22-24^ In four limbs, the RA originated from the medial part of the upper third of the BA and remained along the medial part of the BA until the intercondylar line, after which the RA crossed the median nerve and the BA superficially to reach the lateral part. In the forearm, the course of the RA was normal.^7,12^ Similar to this study, Natsis et al. observed high origin of the RA in the arm in 4 upper limbs.^25^ These investigators also detected the RA beginning from the medial part of the BA above the intercondylar line and crossing the median nerve superficially before following the normal course in the forearm.^25^ In another study, the RA was found arising from the medial part of the BA in the arm.^26^ However, in contrast to the studies mentioned above, in this case, the RA crossed the median nerve twice: firstly, near its origin from the BA in the arm and secondly in the cubital fossa. Subsequently, it followed the normal course in the forearm.^26^ Akin to the study by Pelin et al., the crossing of median nerve twice by a high-origin RA is also described in literature.^27^ Another variant course of the RA, arising from the BA in the arm, was also found.^7^ Here, the authors found the RA arising from the lateral part of the upper BA.^7^ This anomalous RA crossed the median nerve anteriorly, passing downwards along the medial part of the biceps tendon, after which it followed the normal course in the forearm.^7^

Abnormal origin of the RA anomalously related to the median nerve, the BA, and the biceps and brachialis muscles in the arm is also observed.^7^ The RA with a superficial course in the arm may be intimately related to the cephalic vein and so in the absence of this information, medication may be introduced into the RA instead of the cephalic vein, creating complications.^28^ In addition, an abnormal course of the RA at the wrist may create difficulties in palpating the RA at the wrist, resulting in cannulation failure.^9^ With the advent of new diagnostic and surgical techniques, use of the RA has greatly increased in various surgical and radiological procedures like embolization, radial forearm flap in reconstructive surgeries of the arm, as a graft for coronary bypass, and in the transradial approach during coronary interventions.^26^ Moreover, the transradial procedure is the most commonly used technique as compared to the transfemoral or transbrachial procedures for coronary artery related interventions due to the fact that the RA has a superficial course, due to which it is easy to obtain hemostasis, and also because main veins and nerves do not lie in close proximity to the RA.^29^ Not only this, conventional use of the great saphenous vein as a coronary bypass graft is increasingly being replaced by use of the RA, as the latter provides better and longer-term patency.^30^ In addition, aberrant origin and course of the RA may provoke difficulties during measuring blood pressure and interpretation of angiographs, result in puncture of the superficially located RA causing finger gangrene and muscular contraction, or lead to iatrogenic damage to the artery during surgical manipulation in orthopedic, plastic, or vascular surgeries.^21^

A variant RA may compress the median nerve causing neuropathy and physicians may mistake such radiculopathy or neuropathy with those caused by other reasons. As such, precise information about the course of the RA in relation to nearby peripheral nerves is essential during use of vascularized nerve homografts.^31^

Thus, in-depth knowledge of the anomalous path of the RA is very useful to clinicians, especially vascular surgeons and radiologists

### Normal and abnormal branching pattern of the radial artery

Normally, the RA gives off the radial recurrent artery, a dorsal carpal branch, a palmar carpal branch, a superficial palmar branch, and muscular branches in the forearm. Variations in the branching pattern of the RA are mostly observed involving the radial recurrent artery, while other branches of the RA exhibit consistency in origin from the RA.

As per the opinion of Joseph et al.,^21^ variations in the branching pattern of the RA are seldomly described in literature. In one study, the radial recurrent artery was observed to arise from the RA in 83.3% of males and 82.5% of females and from the BA in 15% of cases.^7^ Moreover, the palmar carpal artery was documented to be absent in 22.5% of cases.^7^ However, in 2% of cases, the radial recurrent artery was found to be absent.^7^ In another study by Gupta et al.,^32^ the radial recurrent artery was observed to originate from the BA in 12% of cases and the palmar carpal artery was reported to be absent in 26.7% of cases. Knowledge of variant distributions of both radial and recurrent radial arteries is very useful for achieving safe and successful outcomes in plastic and reconstructive surgery involving the forearm, as the recurrent radial artery is frequently used in forearm free flap surgeries.^7^ In addition, anastomosis using radial recurrent artery vessels is carried out in free radial forearm transplantation for salvage operations.^33^

Thus, in-depth information about variant branching patterns of the RA has great clinical and surgical importance for cardiac catheterization for angioplasty, pedicle flaps, or arterial grafting. Possible anomalous configurations of the RA in terms of branching pattern should be evaluated preoperatively to avoid untoward incidents intraoperatively and postoperatively.^34^

### Variant diameter of the radial artery

Some investigators measured the diameter of the RA,^7,8,11,35^ as documented in Table 2. As is clear from Table 2, the external diameter of the RA in male cadavers was equal in both right and left upper limbs, while in female upper limbs, the mean of the RA external diameter was slightly larger in right upper limbs than in left upper limbs.^7^ However, using ultrasound, Tariq et al. found that the mean diameter of the RA was larger on the right side (2.3 ± 0.4 mm) and in males (2.3 ± 0.39) than on the left side (2.2 ± 0.4 mm) and in females (2.11 ±0.29).^11^ Using angiographic studies, the mean diameter of the RA was found to be 2.6 ± 0.5 mm with a range of 1.6 to 3.8 mm, as described by Yokoyama et al.^8^ In this study, the luminal diameter of the RA at 2 cm proximal to the styloid process was observed to be larger in males than in females^8^ (Table 2).^7,8,11,35^ In a two-dimensional ultrasound study, the mean luminal diameter of the RA was 2.6 ± 0.41 mm at 1–2 cm proximal to the styloid process,^36^ which is comparable to that observed in the angiographic study. In another study using cadavers, the mean diameters of the RA in proximal and distal portions were 2.3 and 2.2 mm respectively.^37^ In addition to this, the mean diameter of the RA in a Japanese population was observed to be 3.1 ± 0.06 in male and 2.8 ± 0.6 mm in female patients,^35^ which is larger in males than females, in confirmation of the aforementioned studies. The disparities in mean values of RA diameters may be due to the different methods (cadavers, angiography, and ultrasound) used to measure mean RA diameters. Detailed information about variant diameters of the RA is very useful for interventional procedures like angiography, graft replacement, catheterization, and bypass surgeries.^7^ Besides being useful to cardiologists, RA diameter is also of utmost use to radiologists for deciding on the size of cannula during transradial coronary procedures and other microsurgical techniques.^7^

**Table 2:** Displaying the diameter of the radial artery.

Author	External/ luminal diameter					
	Right limb of male	Left limb of male	Right limb of female	Left limb of female	Study material	
Nasr^7^	3.3 ± 0.7 mm, 1 cm distal to origin of RA and 3.1 ± 0.7 mm at a distance 2 cm proximal to the end of styloid process	3.3 ± 0.7 mm, 1 cm distal to origin of RA and 3.1 ± 0.7 mm at a distance 2 cm proximal to the end of styloid process	3.07 ± 0.5 mm 1 cm distal to origin of RA and 2.76 ± 0.49 at a distance 2 cm proximal to the end of styloid process	3.02 ± 0.48 mm 1 cm distal to origin of RA and 2.74 ± 0.45 mm at a distance 2 cm proximal to the end of styloid process	Cadaveric study	
Yokoyama et al.^8^	2.69 ± 0.4 mm in males		2.43 ± ± 0.34 mm in females		Angiographic study	range was 1.6 to 3.8 mm
Ashraf et al.^11^	2.3 ± 0.39 mm in males		2.11 ± 0.29 mm in females		-	-
Loh et al.^35^	3.1 ± 0.06 mm in males		2.8 ± 0.6 mm in females		-	-

In addition to the anomalies mentioned above, the RA having a tortuous rather than straight configuration has been reported by some investigators.^12,20,24^ Hassan et al.,^20^ observed extreme RA tortuosity in 2.1%. This morphological anomaly of the RA was found in 1.88% of cadavers by Kadel et al.,^12^. Information about this anomalous RA entity is essential as it may culminate in failure of transradial catheterization^38^ and the RA can even be damaged if transradial catheterization is carried out in an RA with tortuous configuration. In addition to this, RA tortuosity may cause misinterpretation of radiographs.

The radial artery enters the palm between the heads of the first dorsal interosseous muscle and continues as the deep palmar arch.^1^ A superficial branch of the radial artery anastomoses with the superficial branch of the ulnar artery to form the superficial palmar arch. This anastomosis is crucial for supplying blood to the palmar region of the hand and fingers. The superficial palmar branch of the RA was found to be absent in 5% of cases and in 6% of casers it was observed to emerge at a higher level than normal.^7,32^ The normal and variant configurations of the superficial palmar branch of the RA forming the superficial palmar arch are important for surgical interventions in the hand. Thus, Allen’s, test which is a clinical maneuver to assess collateral circulation in the hand, should be performed before invasive procedures, such as arterial puncture or hand surgery.^39^

Allen’s test: During Allen’s test, compression of the radial and ulnar arteries is performed to halt blood flow, followed by releasing compression of one artery while the other is kept compressed. This allows observation of how quickly the color of the hand is restored, indicating the effectiveness of collateral circulation through the superficial palmar arch. These aspects are crucial to ensure safe and successful intervention involving the radial artery and its associated circulation in the hand.

## CONCLUSION

The RA varies immensely in origin, course and branching pattern. The literature search results reveal that nowadays the RA is commonly used for coronary procedures such as coronary artery bypass grafting and transradial catheterization, for pedicle flaps, and for managing radiculopathy and neuropathy in preference to the femoral artery due to its superficial course which makes it easily accessible and easily compressed for hemostasis. Not only this, due to the superficial course of the RA, the patient can be ambulated soon with enhanced postoperative comfort. It is also commonly used for cosmetic surgeries such as forearm flaps, and in renal dialysis for construction of autogenous fistulae. So, knowledge of the normal and anomalous configurations of the RA is essential for vascular, reconstructive, cardiac, and orthopedic surgeons or for radiological manipulation, supporting correct decision-making and facilitating better preoperative evaluation for surgical and radiological interventions, avoiding untoward postoperative results. It is further advised that angiographic or arteriographic pre-evaluation of the origin, course, and branching pattern of the RA should be conducted for the best postoperative results.
